# Les fracture-luxations transolécraniennes

**DOI:** 10.11604/pamj.2015.22.52.7744

**Published:** 2015-09-18

**Authors:** Mohamed Fahsi, Hamza Benameur, Yassir El Andaloussi, Driss Bennouna, Mustapha Fadili, Mohamed Nechad

**Affiliations:** 1 Service de Chirurgie Orthopédique et Traumatologique (Aile IV), CHU Ibn Rochd, Casablanca, Maroc

**Keywords:** Transolécranienne, fracture–luxation, Coude, olécrane, Transolecranon fracture dislocations, fracture dislocations, elbow, olecranon

## Abstract

Les fracture-luxations transolécraniennes sont une entité rare des fracture-luxations du coude. Il s'agit d'une lésion complexe qui peut compromettre le pronostic fonctionnel du coude. Dix patients étaient diagnostiqués dans notre service entre janvier 2005 et novembre 2012. Tous nos patients étaient de sexe masculin, l’âge moyen était de 29 ans. Les fractures de l'olécrane étaient complexes et comminutives dans sept cas et simples chez trois patients. Deux cas étaient associés à des fractures de la tête radiale (Mason III) et deux autres à des fractures de l'apophyse coronoïde. Les résultats étaient évalués après un recul moyen de trois ans et demi par le score de BROBERG et MORREY: trois cas étaient excellents, quatre bons, deux moyens et un mauvais. Cette lésion complexe nécessite une bonne reconstruction de la surface articulaire et une réparation des lésions associées pour permettre une rééducation précoce, seul garant d'une bonne récupération fonctionnelle.

## Introduction

La fracture-luxation transolécranienne est une lésion complexe caractérisée par une luxation de l'articulation huméro-ulnaire associée à une fracture de l'olécrane. Elle constitue une variété rare des fracture-luxations du coude. L'incidence réelle de cette lésion est difficile à estimer en littérature pour plusieurs raisons, qu'on va détaillées dans notre discussion. Beaucoup d’études ont abordé le sujet des fracture-luxations du coude [1–6], mais quelques publications ont été intéressées par cette lésion [7]. Le traitement de ces fracture-luxations est délicat, il nécessite une bonne réduction anatomique et ne doit pas négliger les lésions associées qui pourraient mettre en jeu la stabilité et le pronostic fonctionnel du coude. Le but de cette étude est de préciser les caractéristiques épidémiologiques de cette lésion particulière et de détailler ses modalités thérapeutiques et fonctionnelles.

## Méthodes

**Figure 1:**
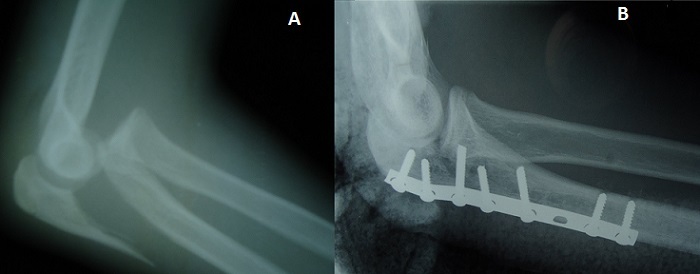
(A) Fracture simple de l'olécrane avec luxation antérieure de l'avant bras; (B) aspect après réduction et ostéosynthèse par plaque

**Figure 2:**
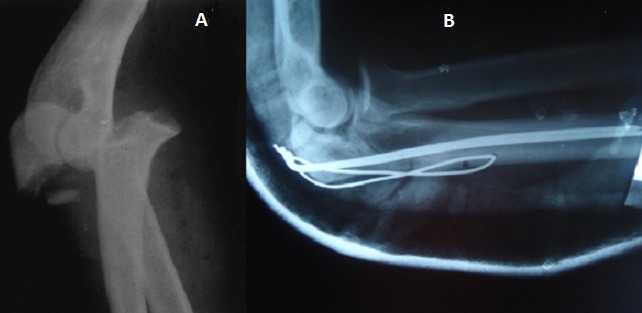
(A) Fracture de la base de l'olécrane avec luxation antérieure de l'avant bras; (B) aspect après réduction et embrochage haubanage

Nous rapportons une étude rétrospective de dix cas de fracture-luxation transolécranienne, colligés entre janvier 2005 et novembre 2012. Le diagnostic était posé par la présence de fracture métaphysaire très proximale de l'ulna associée à une luxation antérieure de l'avant bras dans la radiographie de profil (Figure 1, Figure 2) et sans atteinte de la radio-ulnaire proximale. Tous nos patients ont été suivis à la consultation, interrogés, examinés et contrôlés radiologiquement. La période moyenne de suivi était de 3,5 ans. On a évalué l'activité quotidienne de nos patients ainsi que la reprise de l'activité professionnelle. On a adopté le score de Broberg et Morrey pour l’évaluation des résultats fonctionnels.

## Résultats

L’âge moyen était de 29 ans avec des extrêmes allant de 15 à 66 ans. Tous nos patients étaient de sexe masculin. Parmi eux, 70% des cas étaient des travailleurs manuels. L’étiologie était dominée par les chutes dans 60% des cas, suivies par les accidents de la circulation dans 40% des cas. Le mécanisme retenu chez huit patients était un choc direct sur la partie proximal de l'avant bras, coude fléchi à 90 degrés. Les fractures de l'olécrane étaient complexes dans sept cas, simples dans trois cas et intéressaient la base dans tous les cas. Les lésions associées se résumaient à deux fractures de la tête radiale classées Mason III et deux fractures du processus coronoïde. On n'a pas noté de lésions nerveuses, vasculaires ou d'ouvertures cutanées dans notre série. Nos patients étaient opérés dans un délai moyen de trois jours, tous en décubitus latéral. Nous avons procédé à l'ostéosynthèse comme le décrit (Tableau 1). La rééducation était précoce après une immobilisation de 15 jours. Nous avons relevé un cas de raideur articulaire chez un patient âgé de 66 ans qui présentait une fracture complexe de l'olécrane, associée à une fracture de la tête radiale et qui n'a pas bien entretenu sa rééducation et un cas d'instabilité chronique résiduelle chez un patient qui présentait une fracture de l'apophyse coronoïde et chez qui on a réalisé une résection de la tête radiale. Nous n'avons noté aucun cas de pseudarthrose ou d'ostéome para-articulaire. Nos résultats étaient évalués, après un recul moyen de 3,5 ans, par le score fonctionnel de Broberg et Morrey, trois étaient excellents, quatre bons, deux moyens et un mauvais. Si on considère les résultats excellents et bons comme résultats satisfaisants, 70% de nos patients auraient des résultats satisfaisants. En comparant les résultats en fonction du type de lésions, nous avons constaté que les fractures simples de l'olécrane et les fractures isolées sans lésions osseuses associées donnaient de bons résultats fonctionnels.

**Tableau 1:** Résultats

**Nombre de cas**	10
**Lésions**	
Fracture simple de l'olécrane	3
Fracture complexe de l'olécrane	7
Fracture de la tête radiale	2
Fracture du processus coronoïde	2
**Traitement**	
Plaque visée 1/3 de tube	8
Embrochage haubanage	2
Embrochage de la tête radiale	1
Résection de la tête radiale	1
Vissage de rappel du processus coronoïde	2
**Score Broberg et Morrey**	
Excellent	3
Bon	4
Moyen	2
Mauvais	1

## Discussion

La fracture-luxation transolécraniennes est une lésion rare décrite en 1974 par BIGA et THOMINE [8]. Quelques publications se sont intéressées par cette lésion (Tableau 2) et des auteurs ont rapporté un seul cas dans leurs études [9, 10]. Il s'agit d'une lésion complexe qui traduit un traumatisme de haute énergie [4, 7, 8, 11, 12]. Elle se définie comme une fracture de l'olécrane associée à une luxation antérieure de l'avant bras par rapport à la trochlée humérale sans atteinte de la radio-ulnaire proximal, le ligament annulaire reste intact, ce qui la différencie des lésions de type Monteggia [7, 13, 14]. L'incidence de cette lésion est difficile d'estimer dans la littérature pour plusieurs raisons, notamment l'absence de classification unifié pour les fractures de l'olécrane [5, 7, 12, 13, 15–17]. Mouhsine et al. [7] ont rapporté une incidence de 22% dans leur série, ils expliquent cette faible incidence par une confusion possible avec les lésions de type Monteggia. Ring et al. [14] ont retrouvé dans 70 observations codées initialement comme fracture de Monteggia, 13 cas avaient réellement une fracture-luxation transolécranienne. Bien que dans chaque cas le traitement est une réduction anatomique et stable, le traitement des fractures de Monteggia consiste à une fixation de la diaphyse de l'ulna qui va permettre une réduction, dans la plupart des cas, de la tête radial. Si la luxation de la tête radiale persiste, elle doit être abordée [18]. Pour les fracture-luxations transolécraniennes, le but est de rétablir le contour et la dimension de la grande cavité sigmoïde [4, 14]. D.Ring et al [4, 14] ont décrit que les ligaments collatéraux du coude semblent être épargnés dans cette lésion par rapport à une luxation antérieure pure du coude. D'autre part Mouhcine et al. [7] ont noté une seule étude en littérature dans laquelle une reconstruction ligamentaire était nécessaire pour rétablir la stabilité du coude [12]. Dans notre série aucune réparation ligamentaire n'a été nécessaire.

**Tableau 2:** Séries de la littérature traitant la fracture-luxation transolécranienne

Auteur	Nombre de cas
Wilppula [14]	5
Biga and Thomine [8]	13
Scharplatz and Allgower [5]	3
Guerra and Innao [9]	24
Loup [24]	1
Wolfgang [15]	5
Wilkerson [25]	1
Ring [12]	17
Bailey [13]	11
Ikeda [10]	4
S.M.J. Mortazavi [21]	8
Mouhsine [7]	14
Notre serie	10

L'apophyse coronoïde est incriminée, selon plusieurs auteurs [14, 19–22], dans l'instabilité du coude et l'arthrose post traumatique. Elle doit être fixée de façon solide pour permettre une continuité de la cavité et commencer une rééducation précoce. Certains auteurs préconisent une greffe osseuse s'il y a une comminution entre l'olécrane et le processus coronoïde [14]. Dans notre série, pour les deux cas où il y avait une fracture de l'apophyse coronoïde, on a noté une instabilité chronique résiduelle chez un patient qui présentait une fracture comminutive de la tête radiale et chez qui on a réalisé une résection de la tête. Le traitement de ces lésions repose sur une fixation solide et un bon rétablissement de la congruence articulaire. La majorité des auteurs [14, 15, 23, 24] préconisent la fixation des fractures comminutives de l'olécrane par plaque visée dorsale. Ring et al. [14] préconisent une plaque à compression dynamique (DCP) qu'une plaque 1/3 de tube du fait de ca rigidité insuffisante. Elle était de mise dans huit cas, sept cas de fractures comminutives et dans un cas de fracture oblique de l'olécrane. La fixation par embrochage haubanage peut être suffisante pour certaines des fractures simples de l′olécrane [4]. Chez deux de nos patients, cette méthode a montré son efficacité. Les résultats fonctionnels sont évalués, chez la majorité des auteurs, par le score fonctionnel de Broberg et Morrey [25]. Les résultats satisfaisants dans les séries de littérature varient entre 71 et 88% des cas. Pour notre série les résultats satisfaisants étaient notés dans 70% des cas. Nous n'avons observé aucun cas d'arthrose post traumatique chez nos patients, ceci peut être expliqué par le recul court de suivi dans notre série. La fracture-luxation transolécranienne est moins susceptible d'entraîner une arthrose à condition que le contour et la dimension de la cavité sigmoïde soit restauré. Par conséquent, la prévention de l'arthrose post-traumatique du coude repose moins sur la congruence de la surface de la cavité sigmoïde que sur la restauration de la relation appropriée entre la facette articulaire coronoïdienne, l'olécrane et la trochlée humérale [14].

## Conclusion

Le traitement optimal de fracture-luxation transolécranienne du coude commence par l'identification de la lésion comme une perturbation de l'articulation ulno-humérale, étant souvent confondue avec des fractures de Monteggia. La restauration de la continuité de la cavité sigmoïde doit être anatomique par plaque visée, sans oublier le traitement des lésions associées. La reconstruction anatomique et stable, ainsi qu'une mobilisation précoce permettent d'obtenir de bons résultats fonctionnels.
